# Progress in research on tumor microenvironment-based spatial omics technologies

**DOI:** 10.32604/or.2023.029494

**Published:** 2023-09-15

**Authors:** FANGMEI XIE, NAITE XI, ZEPING HAN, WENFENG LUO, JIAN SHEN, JINGGENG LUO, XINGKUI TANG, TING PANG, YUBING LV, JIABING LIANG, LIYIN LIAO, HAOYU ZHANG, YONG JIANG, YUGUANG LI, JINHUA HE

**Affiliations:** 1 Central Laboratory, Panyu Central Hospital of Guangzhou, Guangzhou, China; 2 Department of General Surgery, Panyu Central Hospital of Guangzhou, Guangzhou, China; 3 Administrating Office, He Xian Memorial Hospital, Southern Medical University, Guangzhou, China

**Keywords:** Spatial omics, Spatial transcriptomics, Spatial proteomics, Tumor microenvironment

## Abstract

Spatial omics technology integrates the concept of space into omics research and retains the spatial information of tissues or organs while obtaining molecular information. It is characterized by the ability to visualize changes in molecular information and yields intuitive and vivid visual results. Spatial omics technologies include spatial transcriptomics, spatial proteomics, spatial metabolomics, and other technologies, the most widely used of which are spatial transcriptomics and spatial proteomics. The tumor microenvironment refers to the surrounding microenvironment in which tumor cells exist, including the surrounding blood vessels, immune cells, fibroblasts, bone marrow-derived inflammatory cells, various signaling molecules, and extracellular matrix. A key issue in modern tumor biology is the application of spatial omics to the study of the tumor microenvironment, which can reveal problems that conventional research techniques cannot, potentially leading to the development of novel therapeutic agents for cancer. This paper summarizes the progress of research on spatial transcriptomics and spatial proteomics technologies for characterizing the tumor immune microenvironment.

## Introduction

Spatial omics technology integrates the concept of space into omics research. The important feature is that it retains the spatial information of tissues or organs while obtaining molecular information [1]. Initially, the research was carried out in the form of bulk biological or molecular research, but the heterogeneity between cells was masked. As a result, single-cell omics, which can reveal the heterogeneity between cells, is the mainstay of research [2]. Single-cell omics also has problems that cannot be solved, such as losing the spatial location information of the cell and the structure of the tissue [3]; thus, spatial omics technology has been developed. The most important feature of spatial omics is that it visualizes changes in molecular information and yields intuitive and vivid visual results [4]. This may be the biggest breakthrough that spatial omics brings to the field of biology.

Spatial omics include spatial transcriptomics, spatial proteomics, and spatial metabolomics [5]. Among them, the application of spatial transcriptomics and spatial proteomics to the field of tumor microenvironment (TME) research has progressed most rapidly. TME refers to the surrounding microenvironment in which tumor cells exist including the surrounding blood vessels, immune cells, fibroblasts, bone marrow-derived inflammatory cells, various signaling molecules, and the extracellular matrix [6]. The occurrence, growth, and metastasis of tumors are closely related to the internal and external environment of tumor cells, including not only the tissue structure, function, and metabolism of tumor cells but also the internal environment of the tumor itself [7]. Tumor cells can change their environment through immunity, secretion, and metabolism. The TME is heterogeneous and plays an important role in tumor occurrence, metastasis, and drug resistance [8].

One of the core issues hindering tumor therapy is the heterogeneity of the TME. Spatially, TMEs in different tumors have different organizations and hierarchies [9]. The hierarchy of the TME is critical for tumor cell fate determination and development, which are coordinated through precise tumor-intrinsic transcriptional regulation and intercellular communication [10]. In response to external stimuli (e.g., chemotherapy), spatial reprogramming is initiated, including antitumor immune regeneration and stromal cell relocation [11]. Therefore, understanding the spatial structure of the TME is crucial for discovering tumorigenesis mechanisms and designing novel therapeutic strategies. Sequencing technology is an important means for studying the TME, with single nuclei RNA sequencing (snRNA-seq) now in wide use [12]. Conventional sequencing is usually based on two-dimensional planar thinking, whereas the spatial sequencing technology provided by spatial omics elevates thinking to three-dimensional space, which can help solve problems that cannot be solved by conventional sequencing [13]. Techniques commonly used to study the TME include spatial transcriptomics and spatial proteomics technologies.

This paper focuses on the research progress of spatial transcriptomics and spatial proteomics technologies for the study of TME. It is expected that spatial omics technology will be widely used for this purpose and will provide a new direction for determining the molecular mechanisms of tumor occurrence and development.

## Spatial Transcriptomics Technology

**Figure 1:**
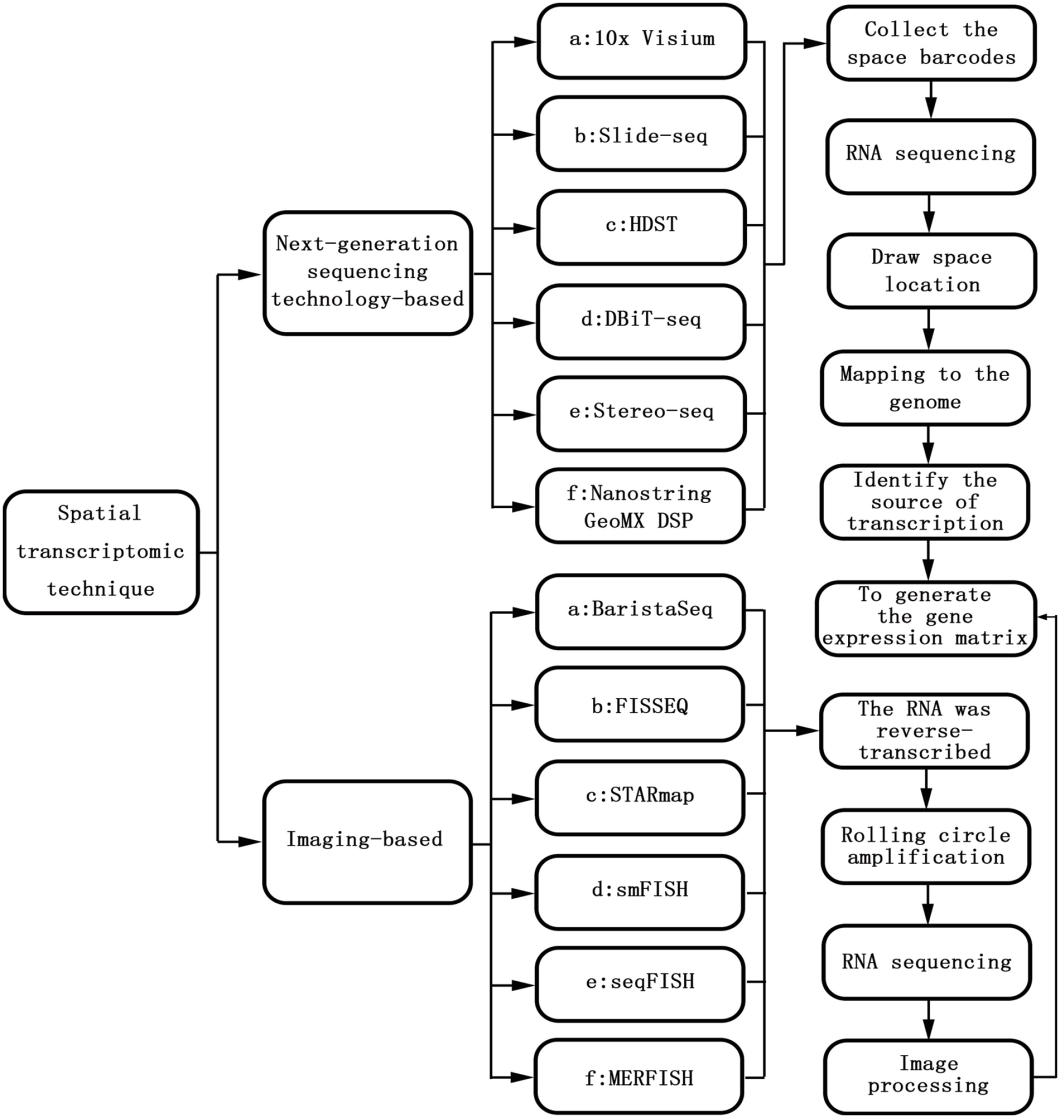
The basic technical principles and classification of spatial transcriptomic techniques.

Gene expression is not only temporally specific but also spatially specific. Both hybrid transcriptomics and single-cell transcriptomics are based on the two-dimensional plane of cells, which reflects mainly the temporal specificity of gene expression [14]. With the continuous improvement of transcription technology and in-depth research, the importance of spatial characteristics has become clear [15]. Spatial transcriptomics (ST) was first proposed in 2016 and is used for visualization and quantitative analysis of the transcriptome at spatial resolution in a single tissue section [16]. Spatial transcriptomics techniques mainly include NGS based techniques (encoding location information onto transcripts prior to NGS sequencing) and imaging based techniques (amplification and sequencing of transcripts in tissues; Imaging probes are hybridized continuously in tissues), and these different techniques can be viewed as converging on a gene expression matrix that captures the transcriptome of each point (that is, a pixel, a cell, or a group of cells). The Basic technical principles and classification of spatial transcriptomic techniques are shown in Fig. 1. The development of spatial transcriptomics can be summarized into two stages. Phase 1: bulk transcriptome (bt) studied-this method can obtain the average gene expression level of a large number of cells, which plays an important role in driving the gene expression of specific cell populations. However, the expression of specific genes in specific cell populations cannot be determined [17]. Phase 2: single-cell nuclei sequencing (snRNA-seq)—this method enables the study of gene expression at the single-cell level and can measure the entire transcription system of a single cell. Specific cell subpopulations within the tissue can also be identified, but because the preparation of single-cell suspensions requires enzymatic dissociation (i.e., disrupting their spatial structure), the spatial location of the sample cannot be obtained [18]. Spatial transcriptome technology utilizes the advantages of both conventional *in situ* and omics technologies, combining the spatial information of mRNAs with morphological content and allowing mapping of where all gene expression occurs. Based on this, a complex and complete gene expression map of the disease can be obtained. Preserving spatial location while identifying distinct cell populations provides important information on the relationship among cellular function, phenotype, and location in the TME [19].

## Spatial Proteomics Technology

With the development of spatial transcriptomics, spatial proteomics technology is also rapidly developing. Spatial proteomics technology can reveal the spatial distribution of proteins in biological tissues to provide a better understanding of the phenotype and activity of cells [20]. Commonly used spatial proteomics technology is based on DSP, which integrates the quantitative information of the proteome with *in situ* tissue information, enabling *in situ* co-analysis of up to hundreds of proteins on a single paraffin tissue section. Unlike multiplexed immunohistochemistry and multiplexed immunofluorescence, spatial proteomics technology captures the target protein *in situ* using an antibody coupled to the nucleic acid probe, followed by the release of the nucleic acid probe through special photodissociation [21]. It uses the nCounter digital label technology for counting and quantification, thereby directly reflecting the abundance of the target protein. Spatial proteomics uses antibody-conjugated nucleic acid to achieve quantification, eliminating the influence of spectral overlap in conventional multiplex analysis and greatly improving the throughput of detectable targets through the unique molecular tag technology of nCounter for counting and quantification. A series of enzymatic reactions such as nucleic acid amplification is not required, which greatly improves the fidelity and accuracy of the data [22].

## Spatial Transcriptomic Technology and Spatial Proteomics Technologies Reveal the TME

Spatial transcriptomics and spatial proteomics technologies can combine histopathology, tumor immunity, and expression profiling. It has strong advantages in TME research, tumor heterogeneity, and tumor immunity research. In recent years, researchers have made some progress in revealing the microenvironment of skin, lung, digestive, breast, blood, and prostate cancers (PCa) by using spatial transcriptomics and spatial proteomics technologies (Table 1).

**Table 1:** Summary of progress in research on spatial transcriptomic and spatial proteomics technology for revealing tumor microenvironment

Tumor type	Technology	Significance
Melanoma	Nanostring GeoMX DSP	To explain TME [23]; discovery of new tumor biomarkers for early diagnosis and prognosis [24,25], screening patients who are more suitable for PD-1 treatment alone, providing new treatment directions [26].
cSCC	Single-cell RNA; sequencing; spatial transcriptomics	To explain TME [27].
NSCLC	Nanostring GeoMX DSP; Nanostring GeoMx Immune pathways	To explain TME, predicting OS and immunotherapy response [28–30].
GC	Nanostring GeoMX DSP	A high-resolution molecular resource of intrapatient and interpatient lineage status across different gastric cancer subtypes is provided [31]; uncovering immunosuppressive myeloid derived suppressor cell function in the gastric tumor microenvironment [32]; SARIFA has the potential to be a promising biomarker associated with tumor-promoting adipocytes in gastric cancer [33].
PDACs	Single-cell RNA sequencing; spatial transcriptomics; Single-cell RNA sequencing; Nanostring GeoMX DSP	New method reveals spatial distribution of cancer cell subsets [34]; discover the immune evasion mechanism of PDAC, revealing, potential combination immunotherapy [35]; to explain TME, uncovering the immunological impact of neoadjuvant therapy [36].
CRC	Spatial transcriptomics and bulk-RNA sequencing; Nanostring GeoMX DSP	ICAF inhibits the growth of antitumor immune cells and is associated with poor patient prognosis, confirmed high PD-L1 expression in immunotherapy patients [37,38].
TNBC	Nanostring GeoMX DSP	To inform future studies of combination immunotherapy in PD-L1(+) patients [39]; Our data provide early insights into the levels of these markers in the TNBC tumor microenvironment and how they relate to chemotherapy response and patient survival [40].
BC	Nanostring GeoMX DSP	Reveal possible prognostic differences or targeting differences among different profiles [41]; α-SMA can be used as a biomarker of trastuzumab resistance, which provides new ideas for solving the problem of trastuzumab resistance [42]; CD45 may serve as a biomarker for early stratification of sensitive tumors during neoadjuvant HER2-targeted therapy and guide subsequent therapy [43]; subtype-specific immune profiles were confirmed [44]; candidate protein biomarkers were identified [45]; patients with early-stage HER2(+) breast cancer may benefit more from immune-targeted therapy than patients with advanced disease [46].
AML	Nanostring GeoMX DSP	To provide and inform personalized immunotherapy targeting IFNγ-dominant AML subtypes [47].
DLBCL	Nanostring GeoMX DSP	Provide a new direction for anti-LAG3 targeted therapy [48].
CTCLs	CODEX multiplexed tissue imaging; RNA sequencing	Provides a new approach that can inform clinical immunotherapy [49].
PCa	Nanostring GeoMX DSP	HDRBT for immune activation in PCa [50]; demonstrated utility of DSP in accurately classifying tumor phenotypes, assessing tumor heterogeneity, and identifying tumor biology involved in metastatic immune composition [51,52].

**1. Skin cancer**

Melanoma is a common malignant tumor. When the tumor develops local invasion, it forms a solid, spatially restricted inhibitory microenvironment along the tumor-stroma boundary. This environment is caused by high concentrations of cytokines that promote the expression of major histocompatibility complex II (MHC II), indoleamine 2,3-dioxygenase 1 (IDO1), and programmed cell death protein 1 (PD-1). Programmed death-ligand 1 (PD-L1) mediates the co-expression of macrophages, dendritic cells, and T cells [23]. PD-L1 is involved in the immunosuppressive response in the TME and is expressed in cluster of differentiation 68-positive (CD68+) cells [24]. The high expression of CD11c in the sentinel lymph nodes of melanoma patients may be associated with distant metastasis [25]. Compared with uveal melanoma (UM), PD-L1 expression is increased in cutaneous melanoma (CM); however, PD-L1 expression is absent in most CM and UM liver metastases. CD163 expression is also increased in CM liver samples. The degree of immune infiltration in CM and UM metastases is similar, and the ratios of depleted CD8 T cells to cytotoxic T cells, total CD8 T cells, and type 1 T helper cells are significantly elevated in UM metastases, indicating improved therapy for metastatic UM [26]. Cutaneous squamous cell carcinoma is the second most common non-melanoma skin cancer, and its progression depends on genetic susceptibility, environmental factors, use of carcinogenic chemicals, and immunosuppressive drugs [27].

**2. Lung cancer**

CD27, CD3, CD4, CD44, CD45, CD45RO, CD68, and CD163 are significantly enriched in the microenvironment of non-small cell lung cancer, whereas CD34, fibronectin, IDO1, lymphocyte-activation protein 3 (LAG3), arginase 1, and phosphatase and tensin homolog are significantly enriched in normal adjacent lung cancer tissues [28]. The expression of CD66b in the lung cancer microenvironment is associated with resistance to immune checkpoint inhibitor therapy, and the high expression of CD66b indicates shortened overall survival (OS) [29]. The expression of integrin subunit alpha M, CD27, and CCL5 is upregulated in lung cancer tissues with CD163+ cell infiltration. In addition, in macrophage-infiltrated lung cancer tissues, the expression of genes related to the M1 phenotype is upregulated, leading to better results from immunotherapy [30].

**3. Malignant tumor of the digestive system**

In the gastric cancer microenvironment, the proportion of plasma cells is significantly increased and affects the new occurrence and development of diffuse tumors while presenting different fibroblast subtypes. The high expression of inhibin beta A-fibroblast activation protein in cells can be used as a predictor of poor clinical prognosis [31]. Patient-derived organoid co-cultured with immune cells can affect the function of immunosuppressive myeloid derived suppressor cells in the gastric TME. The mechanistic target of rapamycin signaling pathway mediates glioma-associated oncogene homolog 1-induced PD-L1 expression in gastric cancer [32]. Stroma AReactive Invasion Front Areas (SARIFA) is expressed in 20% of patients with gastric cancer, and the OS of these patients is significantly reduced. In addition, highly expressed genes in SARIFA-positive cases are associated with triglyceride breakdown and endogenous sterol metabolism, and SARIFA has the potential to be a biomarker associated with promoting tumor adipocyte formation in gastric cancer [33]. The T-cell immunoglobulin and ITIM domain (TIGIT) is an immune checkpoint. In pancreatic adenocarcinoma (PDAC), the CD155/TIGIT axis is critical for maintaining immune escape in PDAC, and TIGIT/PD-1 combined with CD40 agonists have good antitumor responses [34]. The expression of some key immune biomarkers in the PDAC microenvironment correlates with the type of treatment received before surgical resection, and neoadjuvant therapy has an important impact on the immune response of patients with resectable/marginally resectable PDAC [35]. Subpopulations of pancreatic ductal cells, macrophages, dendritic cells, and cancer cells are highly enriched in a small spatial region, and there is co-enrichment of other different cell types in this region [36]. In colorectal cancer (CRC) tissue, inflammatory cancer-associated fibroblasts (ICAFs) are associated with immune inflammation. Antitumor immune cells such as natural killer cells and monocytes are significantly reduced in ICAF-rich clusters, and ICAF may inhibit the growth of antitumor immune cells [37]. Outcome data from a retrospectively collated training cohort at Royal Edinburgh Hospital in the UK (N = 113) were used to create a combined prognosis model, which identified a subset of patients who demonstrated 100% survival over the 5-year follow-up period. The data used in the model were lymphocyte infiltration,the number of T and B lymphocytes in the 50-micron range, and the CD68+/CD163+ macrophage ratio. The finding was confirmed in a subsequent independent validation cohort that included patients treated in Japan and Scotland (N = 117) [38].

**4. Breast cancer**

PD-L1 is an important potential target for triple-negative breast cancer (TNBC)-targeted therapy. A previous study reported that the RNA profile of PD-L1+ TNBC showed increased dendritic cell, macrophage, and T and B lymphocyte subsets as well as decreased inhibitory cells of bone marrow origin [39]. Compared with PD-L1-TIMEs (tumor-immune microenvironments), PD-L1+ stromal and intraepithelial TIMEs were highly enriched in IDO-1, HLA-DR, CD40, and CD163 and also showed spatially specific changes in CTLA-4, interferon gene stimulator (STING), and fibulin. Macrophage and antigen-presentation-related proteins were the most correlated with PD-L1 [39]. Also, in another TNBC study, it was shown that the high expression of estrogen receptor alpha (ER-α) is associated with longer OS, whereas MART-1 (Melanoma-associated antigen recognized by T cells) expression is associated with shorter OS [40]. The expression of B7 homolog 3 (B7-H3; CD276-encoded) differs significantly in breast cancer patients according to race and is lowest in Black women, whereas the expression of CD8, CD25, CD56, CD127, EpCAM, ER, and Ki-67 correlates with survival prognosis [41]. In a study of resistance to trastuzumab in patients with early-stage human epidermal growth factor receptor 2-positive (HER2(+)) breast cancer, it was found that the high expression of alpha smooth muscle actin (a-SMA) in leukocytes and stromal compartments is associated with shorter DFS; a-SMA can be used as a new biomarker of trastuzumab resistance, providing new ideas for solving the problem of trastuzumab resistance [42]. Another study in HER2(+) breast cancer showed that CD45 can be used as a biomarker to guide the stratification of tumors sensitive to neoadjuvant-targeting HER2(+) therapy and guide subsequent therapy [43]. Different subtypes of breast cancer can be identified using DSP technology; for example, regulatory T cell markers (CD4, CD25, and FOXP3) are highly expressed in basal-like and luminal breast cancers [44]. Major histocompatibility complex II expression and tumor-infiltrating lymphocytes are important prognostic factors in TNBC patients. TNBC patients with long-term disease-free survival (DFS) have higher human leukocyte antigen DR (HLA-DR) expression than relapsed patients, whereas HLA-DR expression in the epithelial compartment correlates with CD4 and inducible costimulatory expression in the stromal compartment of the same tumor [45]. The median tumor-infiltrating lymphocyte (TIL) category in brain metastases was 5% (1%–70%), and the TIL percentage in primary breast tumor tissue and the number of CD4/CD8/Foxp3-positive cells were significantly higher than in brain metastatic breast tumor tissue, especially in patients with triple-negative breast cancer, where reduced TIL number was associated with shorter overall survival [46].

**5. Myeloid leukemia and diffuse large B-cell lymphoma**

The resistance of acute myeloid leukemia (AML) to chemotherapy drugs has always been a difficult clinical problem. Interferon gamma (IFNγ)-related mRNA expression profiles predict both chemoresistance in AML and response to flutuzumab immunotherapy. This provides clues for personalized immunotherapy for IFNγ-dominant AML subtypes [47]. LAG3 is upregulated in diffuse large B-cell lymphoma. The expression of LAG3 in T cells and tumor-associated macrophages (TAMs) was significantly higher than that of CD201 in B cells. It is also expressed in a subset of malignant B cells in TAM-rich regions [48]. The functional immune status of the TME is influenced by factors other than immune cell counts for the treatment of cutaneous T-cell lymphoma with an immunotherapy drug targeting PD-1. SpatialScore was consistent with differences in functional immune status, T-cell function, and tumor cell-specific chemokine recruitment in the TME. This provides new information for clinical immunotherapy [49].

**6. PCa**

PCa usually has a high immunosuppressive microenvironment. Investigators found increased densities of CD4 T cells, CD68+ macrophages, and CD68+ CD11c+ dendritic cells in response to high dose-rate brachytherapy (HDRBT). HDRBT induces significant changes in associated immune cells, and HDRBT converts ‘cold’ PCa tissue into more immune-activated ‘hot’ tissue [50]. Nanostring GeoMX DSP technology is used to quantify transcript and protein abundance in different spatial regions of metastatic PCa (mPCa). The results have shown that most metastases have no obvious inflammatory infiltration and lack expression of PD1, and cytotoxic T-lymphocyte associated protein 4. However, the B7-H3/CD276 immune checkpoint protein is highly expressed in mPCa with high androgen receptor activity, and DSP technology has potential in assessing tumor heterogeneity and identifying immune components in metastases [51]. Using Nanostring GeoMX DSP technology to distinguish among different pathological subtypes of PCa, the results showed that in blast cell typing, immune cells are rich in phosphorylated signal transducer and activator of transcription 3 (STAT3) and Janus kinase/STAT expression. In oncolytic skin lesion typing, immune cells were rich in phosphoinositide 3-kinase (PI3K)/AKT and PI3K/AKT expression [52].

## Conclusions and Prospects

With the advancement of research, the requirements for high-dimensional spatial profiles that can provide valuable information will increase. Spatial transcriptomics technology has recently emerged as a powerful tool to measure spatially resolved gene expression directly in tissue sections, revealing cell types and their dysfunctions in unprecedented detail. Spatial omics can help us to understand the composition of human tissues and the occurrence and development of tumors from a new perspective. However, the commercialization of spatial omics has only recently been completed, and its application is still in its infancy. Despite cutting-edge analytical tools, spatial transcriptomic and spatial proteomic techniques remain flawed. For example, the requirements for the proband of the researcher’s pathological direction are relatively high, and a certain pathological research foundation is required. There is also macro control of valuable research areas. Failure to accurately localize the tissue area under study can lead to deviations in detection results from expectations. Meanwhile, spatial transcriptomic and proteomic techniques require a number of cells in the circled region. RNA detection requires a region of interest of no less than 100 cells (50 can also be successful), and protein detection requires at least 20 cells (at least 10 cells have been tested successfully), and thus single-cell level circle selection and detection cannot be achieved [53]. At present, most of the spatial transcriptome and spatial proteome technologies are still used in basic research. Due to the complexity of the workflow, they cannot become a standard tool for clinical detection like immunohistochemistry. Each technology has certain advantages and disadvantages. Researchers are also working hard to address the technical limitations. At present, the CosMx SMI system has been launched, which can perform ultra-high-resolution imaging of more than 1,000 RNAs and more than 100 protein molecules in tissue sections at the single-cell level and subcellular level, helping researchers achieve accurate and thorough biological analysis [54]. In addition, GeoMX technology has realized simultaneous detection of RNA and protein on a single slice abroad, which is undoubtedly a boon for researchers whose samples are relatively precious [54]. China is also actively carrying out localization. While spatial transcriptomic and spatial protein technologies are applied to basic research, these technologies may become important tools for personalized medicine; for example, in some tissues, a locally acting immune response may be important for thermostatic. When these technologies are applied to clinical work, the complexity of the workflow needs to be solved. Creating a comprehensive and publicly available database as well as establishing separate analysis tools and standardized processes are clinical technology issues that need to be considered and solved in the future. Spatial omics has made great progress in exploring the TME and has great research value. It is not difficult to foresee that with the development of technology, people will have access to high-quality data with higher resolution and wider coverage, which will undoubtedly contribute to the further development of the life sciences.

## Data Availability

Not applicable.

## Abbreviations

scRNA‑seq: Single‑cell RNA sequencing
ST: Spatial transcriptomics
MERFISH: Multiplexed error-resistant fluorescence *in situ* hybridization
FISSEQ: Fluorescence *in situ* sequencing
ExSeq: Expansion sequencing
HDST: High-definition spatial transcriptomics
ER: Estrogen receptor
EpCAM: Epithelial cell adhesion molecule
NGS: Next-generation sequencing
NS: Normal skin
CAFs: Cancer-associated fibroblasts
ICAFs: Inflammatory cancer-associated fibroblasts
Slide-seq: slide-sequencing
DBiT-seq: Microfluidic Barcoding in Tissue Sections-sequencing
Stereo-seq: Spatio-Temporal Enhanced REsolution Omics-sequencing
Nanostring GeoMX DSP: Nanostring GeoMx Digital Spatial Profiler
BaristaSeq: Barcode in situtargeted sequencing
STARmap: Spatially resolved Transcript Amplicon Readout Mapping
smFISH: single-molecule fluorescence *in situ* hybridization
DSP: Digital spatial profiling
DAMP: Damage-associated molecular pattern
OS: Overall survival
SARIFA: Stroma AReactive Invasion Front Areas
TIGID: T cell immunoglobulin and ITIM domains
PAC: Pancreatic adenocarcinoma
TNBC: Triple-negative breast cancer
TIME: Tumor immune microenvironment
B7-H3: B7 homolog 3
MHC: Major histocompatibility complex
HLA-DR: Human leukocyte antigen DR
a-SMA: Alpha-smooth muscle actin
DFS: Disease-free survival
pCR: Pathological complete response
Treg: Regulatory T cell
AML: Acute myeloid leukemia
DLBCL: Diffuse large B-cell lymphoma
LAG3: Lymphocyte activation gene 3
PCa: Prostate cancer
HDRBT: High dose-rate brachytherapy
AR: Androgen receptor
TIS: Tumor inflammation signature
BM: Brain metastases
IDO1: Indoleamine 2,3dioxygenase 1
CD: Cluster of differentiation
CTCLs: cutaneous T-cell lymphomas
